# Conservative Nonhormonal Options for the Treatment of Male Infertility: Antibiotics, Anti-Inflammatory Drugs, and Antioxidants

**DOI:** 10.1155/2017/4650182

**Published:** 2017-01-09

**Authors:** Aldo E. Calogero, Rosita A. Condorelli, Giorgio Ivan Russo, Sandro La Vignera

**Affiliations:** ^1^Department of Clinical and Experimental Medicine, University of Catania, Catania, Italy; ^2^Department of Surgery, Urology Section, University of Catania, Catania, Italy

## Abstract

The nonhormonal medical treatment can be divided into empirical, when the cause has not been identified, and nonempirical, if the pathogenic mechanism causing male infertility can be solved or ameliorated. The empirical nonhormonal medical treatment has been proposed for patients with idiopathic or noncurable oligoasthenoteratozoospermia and for normozoospermic infertile patients. Anti-inflammatory, fibrinolytic, and antioxidant compounds, oligo elements, and vitamin supplementation may be prescribed. Infection, inflammation, and/or increased oxidative stress often require a specific treatment with antibiotics, anti-inflammatory drugs, and/or antioxidants. Combined therapies can contribute to improve sperm quality.

## 1. Introduction

The nonhormonal medical treatment has a relevant role in male infertility since it may solve the cause of infertility, whereas in some other times it may ameliorate sperm parameters by improving the environment where spermatozoa are produced and mature. The nonhormonal treatment may be classified into empirical and nonempirical.

## 2. Empirical Treatment

The empirical nonhormonal medical treatment has been prescribed to patients with idiopathic oligoasthenoteratozoospermia (OAT), patients with OAT due to a noncurable disease, and normozoospermic infertile patients without identifiable risk factors for infertility. Sometimes, a slight improvement of the sperm leads to the achievement of pregnancy.

The scientific evidence of the effectiveness of such a treatment has been scanty. Anti-inflammatory, fibrinolytic, and antioxidant compounds and vitamin supplementation are used. Antioxidant treatment for subfertile couples has been also prescribed to the male partner of couples undergoing assisted reproduction techniques (ART). Further studies are required to clarify the role of these drugs [1].

## 3. Nonempirical Treatment

A nonempirical treatment has been prescribed when the cause of infertility has been identified and it has been curable. Such a treatment has been based on drugs that can specifically eradicate the etiopathogenic noxa. Aside from endocrine causes, three often coexisting conditions interfering with the reproductive function may require nonempirical treatment: infections, inflammation, and/or increased oxidative stress.

A nonempirical treatment for infertility due to urogenital tract infections has been based on the use of specific antibiotics, following identification of the microorganisms by appropriate microbiological investigation and the relative antibiogram. We can distinguish the presence of microorganisms, therefore, as microbial or inflammatory forms.

Microbial forms show the growth of more than 10^3^ pathogenic bacteria or more than 10^4^ nonpathogenic bacteria per ml, in culture of diluted seminal plasma. Some Gram-negative bacteria (Enterobacteriaceae such as* Escherichia coli*,* Klebsiella* species,* Proteus*,* Serratia*, and* Pseudomonas* species) and etiological agents of sexually transmitted diseases (*Chlamydia trachomatis*,* Ureaplasma urealyticum*,* Treponema pallidum*,* Neisseria gonorrhoeae*, etc.) are recognized as “certain pathogens” of the prostate (category II according to the National Institutes of Health classification). On the other hand, some microorganisms of the prostate, which are occasionally detectable in the urogenital tract, are considered by some authors to be “nonpathogenic,” “likely pathogens,” “occasional pathogens” (Gram-positive germs, such as* Enterococcus *spp.,* Staphylococcus aureus*, and obligate anaerobes), or “possible pathogens” (coagulase-negative germs, such as* Staphylococcus haemolyticus*,* Staphylococcus epidermidis*, and mycoplasmas) [2].

Recently, an elevated frequency of HPV infection in patients with infertility [3], as a viral form, cannot rely on antibiotic treatment. The most frequently used families of antimicrobial drugs for the treatment of the microbial forms are fluoroquinolones, macrolides, and tetracyclines [4, 5].

The inflammatory forms are characterized by leukocytospermia (seminal fluid leukocyte concentration > 10^6^/mL) and/or overproduction of reactive oxygen species (ROS). An increased number of leukocytes in the seminal fluid may persist even after antibiotic treatment for a microbial form in some patients with complicated infection of the male accessory glands, such as prostatovesiculoepididymitis (PVE). In addition to leukocytospermia [6], these patients have often abnormal conventional sperm parameters (concentration, motility, and morphology) [7] and other signs of inflammation. Seminal leukocytes are predominantly polymorphonuclear leukocytes (neutrophils), but histochemistry, the technique used for their identification (based on the presence of peroxidase in granulocytes), does not detect other types of leukocytes. Other markers of inflammation are represented by some cytokines (IL-1, IL-2, IL-6, IL-8, IL-18, *α*TNF, and *γ*INF) [8]. In these patients an anti-inflammatory treatment may be done using nonsteroidal anti-inflammatory drugs (NSAIDs) [9] that can be administered simultaneously before or after the antibiotic therapy [10] or nutraceutical compounds with anti-inflammatory and fibrinolytic properties.

The oxidative stress is another important mechanism that damages spermatozoa. It arises from an elevated production of ROS, byproducts of aerobic life, which exceeds the natural scavenger ability of spermatozoa and of the seminal fluid. In fertile men, ROS production and the total antioxidant capacity remain in balance [15]. However, infections, autoimmune disorders, chronic disease, advanced age, alcohol consumption, cigarette smoking, stress, and obesity alter this balance and increase the oxidative stress [16]. In small amount, ROS play a physiological, useful role in sperm function. Spermatozoa produce a small amount of ROS in their earliest stages of development [17]. In this phase, ROS are involved both in the process of sperm chromatin condensation and in the induction of apoptosis or proliferation of spermatogonia, in order to regulate the final number of germ cells [18]. Instead, in mature spermatozoa, ROS are necessary to boost the capacitation process and the acrosome reaction and they are involved in mitochondrial sheath stability and sperm motility. It has been found that spermatozoa with abnormal morphology, mainly those with cytoplasmic residues which indicate their immaturity and reduced fertile potential, produce higher amount of ROS than normally shaped spermatozoa [19, 20].

The seminal plasma contains natural antioxidant, such as vitamins C and E, superoxide dismutase (SOD), glutathione, uric acid, and the polyamine spermine that acts as a free radical scavenger [21]. However, the high concentration of unsaturated lipids in the plasma membrane and the relative paucity of oxyradical scavenger enzyme (due to the virtual absence of cytoplasma) make mature spermatozoa particularly susceptible to oxidative stress. Superoxide anion radical (O_2_^−^) is the main ROS produced by spermatozoa, which generates hydrogen peroxide spontaneously or following SOD activity. The pathways that contribute to ROS production are the NADPH oxidase system at level of cell plasma membrane and the NADH oxidoreductase at the mitochondria level [22].

Infections and/or inflammation of the urogenital tract may increase the number of seminal fluid leukocytes, and their activation results in a ROS overproduction. In fact, leukocytes, under physiological conditions, produce up to 1000 times more ROS than spermatozoa. This production plays an important role in the cellular defense mechanism against infections and inflammation but at the same time may damage spermatozoa. An increased number of leukocytes in the seminal plasma can also be present in varicocele, long sexual abstinence, or exposure to environmental factors [23–28].

The polyunsaturated fatty acids of sperm plasma membrane are a target of ROS action leading to lipid peroxidation, measurable as malondialdehyde (MDA) in the seminal plasma, and loss of sperm motility [21]. ROS can also alter biofunctional sperm parameters, resulting in a greater number of spermatozoa with fragmented DNA that seems to be inversely correlated with sperm count, morphology, motility, and fertilization rate [21, 22]. ROS overproduction causes also a greater frequency of single- and double-strand DNA breaks and increased DNA protein cross-linking [8]. Furthermore, ROS have the ability to damage mitochondrial DNA [21].

These findings support the administration of molecules with antioxidant activity in patients with oxidative stress-induced sperm damage. Antioxidants are widely available and relatively inexpensive when compared to other molecules used for fertility treatment. In addition, there is no evidence of adverse events and they seem to be effective in improving sperm parameters and in increasing a couple's chance of having a live birth [1].

## 4. Antibiotic Treatment

The choice of the antibiotic should be based on the nature of the microorganism identified and the results of the relative antibiogram, since a targeted therapy is recommended. Several classes of antibiotics may be used [29, 30]. We would also point out that most of these microorganisms may be sexually transmitted and it is necessary to stop sexual contacts for the time of treatment and also treat female partner to eradicate the infection when necessary.

### 4.1. Quinolones

Quinolones (ciprofloxacin, levofloxacin, ofloxacin, norfloxacin, pefloxacin, enoxacin, fleroxacin, lomefloxacin, and other new ones) have a favorable pharmacokinetic profile, with excellent oral bioavailability (greater than 95%) for most. However, differences in peak serum concentrations and *β*-half-lives of elimination exist. As suggested by high apparent volumes of distribution and low binding to serum proteins, penetration into many body tissues and fluids is advantageous.

Oral bioavailability of quinolones has been shown to be in general good in sick as well as healthy subjects but is reduced by coadministration with magnesium- and aluminum-containing acids, sucralfate (which contains aluminum), or ferrous sulfate [31]. Quinolones have an excellent ability to penetrate into the prostate and good activity against typical and atypical pathogens. They are associated with phototoxicity, CNS adverse events (variable among the different agents), and tendonitis.

Quinolones are considered a first-line therapy. Among the different quinolones, the most used in clinical practice are ciprofloxacin and levofloxacin. Ciprofloxacin is absorbed primarily on the duodenum and jejunum. Studies also suggest this drug is cleared by transepithelial elimination into the bowel lumen as well as by the renal route. Levofloxacin seems to offer advantages over ciprofloxacin for bacterial eradication rates and clinical improvement in patients with chronic prostatitis [32], whereas another study of similar size and design showed no significant differences between these two agents [11].

Dose and duration should be sufficient to eradicate the infection, for example, ciprofloxacin 500 mg (once or twice/day) and levofloxacin 500 mg (once/daily) for 20–28 days. The treatment may be split into two cycles of 10–14 days, separated by an interval of one-two weeks.

### 4.2. Trimethoprim

Trimethoprim is rapidly absorbed from the gastrointestinal tract, widely distributed into body tissues and fluids, including seminal fluid and prostatic tissue. It is eliminated by glomerular filtration and tubular secretion in urine; only small amounts are excreted in feces by biliary elimination. Trimethoprim is active against many relevant pathogens but has no activity against* Pseudomonas*, some enterococci, and some Enterobacteriaceae. Monitoring is unnecessary. It is a second-line therapy.

Dose and duration should be sufficient to eradicate the infection, for example, 200 mg once or twice/day for 28 days. The treatment may be split into two cycles of 10–14 days, separated by an interval of one-two weeks.

### 4.3. Tetracyclines

Tetracyclines are readily absorbed and bound with plasma proteins, concentrated by the liver in the bile, and excreted in the urine and feces at high concentrations in a biologically active form. These drugs are contraindicated in renal and liver failure. They have good activity against* Chlamydia trachomatis* and Mycoplasma and lower efficacy against coagulase-negative staphylococci,* Escherichia coli*, other Enterobacteriaceae, and enterococci. There is no activity against* Pseudomonas aeruginosa*. They are associated with risk of skin sensitization. Tetracyclines are a second-line therapy. Dose and duration should be sufficient to eradicate the infection.

The* Ureaplasma* sp. isolates are susceptible to doxycycline and josamycin. Most studies report lower resistance rates for tetracyclines (<5%).

Doxycycline is administered at the dose of 100 mg once or twice/day for 28 days. The treatment may be split into two cycles of 10–14 days, separated by an interval of one-two weeks.

### 4.4. Macrolides

Macrolides have good penetration into the prostate and are active against* Chlamydia trachomatis* and Gram-positive bacteria but have an unreliable activity against Gram-negative bacteria. Macrolides are used for special indications, based on the microbiological findings. Minor side effects of macrolide administration include nausea, vomiting, diarrhea, and ringing or buzzing in the ears (tinnitus). Serious side effects, including allergic reaction and cholestatic hepatitis (inflammation and congestion of bile ducts in the liver), are generally associated only with the use of erythromycin.

Dose and duration should be sufficient to eradicate the infection. Azithromycin is prescribed at the dose of 1 g once/day for 7–10 days.

A number of studies have compared the microbiological eradication rate between the different antibiotics.

Higher eradication rates (>90%) have been reported with azithromycin and levofloxacin either alone, in combination, or sequentially, depending upon the infection site (urethral, prostatic, or both) in patients with chronic bacterial prostatitis by* Chlamydia trachomatis* infection [33].

### 4.5. *β*-Lactam Antibiotics

*β*-lactam antibiotics (penicillin derivatives, cephalosporins, monobactams, and carbapenems) have a limited use in male infertility. Some of them can be used if a particular quinolone-resistant male urogenital infection is present, such as acute prostatitis caused by extended-spectrum *β*-lactamase-producing bacteria, which seems to be sensible to imipenem, cefoxitin, and amoxicillin/clavulanic acid [34].

Other uses include management of* Neisseria gonorrhoeae* infections whose prevalence has grown over the past decades. It is the second most commonly reported sexually transmitted bacterial infection in the United States, after* Chlamydia trachomatis*. The 2015 Centers for Disease Control and Prevention guidelines recommend dual therapy with intramuscular ceftriaxone and oral azithromycin as first-line treatment, although components of this type of treatment are met with a high level of resistance [35].

The recommended dose for uncomplicated gonococcal urethral infections is a single dose of ceftriaxone, 250 mg intramuscularly, and either azithromycin (1 g orally) or doxycycline (100 mg orally twice/day) for seven days.

### 4.6. Antibiotics and Sperm Toxicity

Although the antibiotic treatment has been essential to preserve or restore normal sperm parameters in urogenital infections, some of them have spermotoxic effects. This side effect has not yet been directly shown in humans by randomized clinical trials, but there are data concerning testicular and/or sperm toxicity for some antibiotics in rats or mice. These include ciprofloxacin and pefloxacin [36], ofloxacin [37], lomefloxacin [38], tetracyclines [39], cefonicid and other cephalosporins [40], and norfloxacin in quails [41].

## 5. Anti-Inflammatory Treatment

### 5.1. Nonsteroidal Anti-Inflammatory Drugs (NSAIDs)

#### 5.1.1. Salicylates

Salicylates include salicylates, diflunisal, and salsalate. There are no available data on the last two categories. The negative effect of mesalazine on fertility has been shown in patients with irritable bowel disease that at a certain point requires treatment cessation to achieve fertility. Indeed, the mean sperm concentration, sperm motility, percentage of spermatozoa with normal form, seminal fluid volume, and total motile sperm count increase after mesalazine discontinuation [42]. Another study showed a decrease of sperm count and motility in mice treated with sulfasalazine [43]. The administration of salicylate (650 mg four times a day) significantly decreased sperm motility after 3 days of treatment in four patients. The negative effect on sperm motility was not due to necrozoospermia [44].

#### 5.1.2. Fenamic Acids

No data on fenamic acids are available on male fertility.

#### 5.1.3. Profens

Few data are available on profens. A study showed that ibuprofen may cause a significant alteration of sperm parameters and chromatin/DNA integrity in mice. These deleterious effects are dose-dependent and are observed in early and late stages of drug administration [45]. On the other hand, another study showed that reproductive damage induced by continuous or intermittent hypoxia was partially ameliorated by the simultaneous treatment with ibuprofen [46].

#### 5.1.4. Cox-2 Inhibitors (Coxibs)

The data are contrasting for this class of NSAIDs. Studies on mice and turkeys showed a negative effect of coxibs on sperm parameters [47, 48]. In contrast, sperm parameters (but not morphology) improved in patients with chronic pelvic pain syndrome treated with a combination of *α*-blockers and coxibs [49]. Similarly, sperm motility and morphology improved and seminal fluid leukocyte concentration decreased in patients with amicrobial leukocytospermia undergoing assisted reproductive technique treated with rofecoxib [50]. Similar data were reported in another study whose patients were given valdecoxib [9]. These findings suggest that coxibs may be prescribed in infertile patients with leukocytospermia. However, more data are needed to assess this indication.

The dose used in the clinical trials was of 25 mg for rofecoxib, for a period of 30 days or 20 mg once/day for valdecoxib for two weeks. This indication has been currently off-label for leukocytospermia in Italy.

#### 5.1.5. Arylacetics

No recent data are available on the effects of arylacetics for male fertility.

#### 5.1.6. Sulfonanilides

Sulfonanilides include the relatively COX-2 selective nimesulide. A study was conducted with nimesulide, in 30 cases of abacterial prostatovesiculitis and no specified infertility issue, showing that its oral administration at the dose of 100 mg twice/day for three cycles of 10 days each reduced dysuric symptoms and improved inflammatory signs at transrectal prostate ultrasound evaluation. However, no statistically significant changes on sperm count and motility were observed, while a significant reduction in the number of abnormal forms occurred [51]. Another study showed that nimesulide does not seem to be spermatoxic in prepubertal rats at normal therapeutic doses [52].

#### 5.1.7. Oxicams

No data are available on the effects of oxicams on male fertility.

Overall, NSAIDs should be considered for the treatment of the acute forms of male accessory gland inflammation for symptoms relief and they should be avoided for chronic usage (if possible) in patients with infertility. Additional studies are needed to explore the possibility of positive effects of coxibs on patients with “idiopathic” leukocytospermia.

### 5.2. Steroidal Anti-Inflammatory Drugs

Glucocorticoids are employed for the treatment of infertility when antisperm antibodies (ASA) are demonstrated. Recent studies have investigated the effects of ASA and the correlation between ASA and sperm parameter abnormalities, but inconsistent results have been reported. A recent meta-analysis shows a significant negative effect of ASA on sperm concentration and total sperm motility (progressive + nonprogressive) which are lower in ASA positive patients than in ASA negative and sperm liquefaction which has been longer in ASA positive patients [53]. In addition, an immune-suppressive treatment has been found poorly effective and many other treatments have been proposed such as ART (intrauterine insemination and in vitro fertilization) and laboratory techniques (sperm washing, immunomagnetic sperm separation, proteolytic enzyme treatment, and use of immunobeads) [54].

## 6. Fibrinolytic Treatment

In this section, we discuss three of the main fibrinolytic agents evaluated in literature and widely used clinical practice: serratiopeptidase, bromelain, and escin.

### 6.1. Serratiopeptidase

Serratiopeptidase is a proteolytic enzyme of 45,000–60,000 kD molecular weight and particularly a metalloprotease containing a zinc atom which plays a pivotal role for its proteolytic activity [55]. It derives from nonpathogenic enterobacteria belonging to* Serratia* species E-15. This microorganism was originally isolated during late 1960s from the intestines of silkworm* Bombyx mori*, its natural habitat. The enzyme facilitates the emerging moth to dissolve its cocoon. Serratiopeptidase is produced by purification from culture of* Serratia* E-15 bacteria [56].

In rats, orally administered serratiopeptidase is absorbed from the intestinal tract and reaches the circulation in an enzymatically active form [57, 58]. However, pharmacokinetic data including its oral bioavailability and the minimal concentration required for its therapeutic action are not reported in humans.

Serratiopeptidase seems to act as analgesic, anti-inflammatory, and fibrinolytic/caseinolytic [59–61]. As for the anti-inflammatory property, this enzyme reduces swelling by decreasing the amount of fluid in the tissues, thinning the fluid, and facilitating fluid drainage. In addition, its enzyme activity dissolves dead tissue surrounding the injured area so that healing is accelerated. It acts also by modifying cell-surface adhesion molecules that guide inflammatory cells to site of inflammation. Its fibrinolytic/caseinolytic activity relates on the breaking down of fibrin and other dead or damaged tissues without harming the healthy tissue. This could enable the dissolution of blood clots, atherosclerotic plaques, and facilitating antibiotic penetration. Two animal and eight clinical studies support its use as anti-inflammatory agent but larger, better-designed, placebo-controlled trials are needed to clearly prove its efficacy. In addition, data on tolerability and long-term safety of this enzyme lack [62].

A clinical study showed a small significant increase in sperm count compared to pretreatment value, after treatment with levofloxacin plus serratiopeptidase, in patients with >2 years infertility and male accessory gland infection/inflammation, while sperm motility and morphology did not change [63]. It is possible that the enzyme could enhance the penetration of antibiotics in the infected tissue and increase the activity of quinolones against the development of bacterial biofilms [64].

There are not many published articles on the adverse drug reactions (ADRs) to serratiopeptidase. The only information may be obtained from drug company monographs. The ADRs include allergic skin reaction that ranges from dermatitis to extreme cases of Stevens-Johnson syndrome or erythema multiforme, muscle aches and joint pains, gastric disturbances like anorexia, nausea, and abdominal upset, cough rarely pneumonitis [65], and coagulation abnormalities. It has to be taken on an empty stomach or at least two hours after eating, and no food should be consumed for about 30 minutes after the ingestion of serratiopeptidase.

The recommended dose of serratiopeptidase for specific indications, in particular, has been not mentioned anywhere; however, serratiopeptidase-based drugs commonly range from 30000 to 60000 UI/day.

### 6.2. Bromelain

Bromelain has been studied since 1875 and it is used as a phytomedical compound [66]. Bromelain is a mixture of different thiol endopeptidases and other components like phosphatases, glucosidase, peroxidases, cellulases, glycoproteins, carbohydrates, and several protease inhibitors [67]. Bromelain seems to exert a wide range of therapeutic benefits; it is known to enhance absorption of drugs, particularly antibiotics [68, 69]. Bromelain acts on fibrinogen giving products that are similar, at least in its effect, to those formed by plasmin [70]. Experiment in mice showed that antacids such as sodium bicarbonate preserve the proteolytic activity of bromelain in the gastrointestinal tract [71]. Bromelain is absorbed by the human intestine without degradation and loss of its biological activity [72, 73]. The body can absorb significant amount of bromelain; about 12 g/day of bromelain can be consumed without any major side effects.

Clinical studies have shown that bromelain may be useful for the treatment of several disorders (cardiovascular diseases, osteoarthritis, immunogenicity, blood coagulation, fibrinolysis, diarrhea, effects on cancer cells, and debridement burns) [74]. No data are however available on the role of bromelain given alone on male fertility. However, many studies suggest an effect of bromelain on antibiotic potentiation; combined bromelain and antibiotic therapy has been shown more effective than antibiotic alone in eradication of various infections, including urinary tract infection [75].

Bromelain has been used in therapeutic schemes ranging from 160 mg per day to 750–1000 mg/day. The best results occurred when it was administered four times a day and the effectiveness was dose-dependent [76]. Bromelain has very low toxicity [77]. In human clinical tests, side effects were generally not observed; however, a report indicated that individuals with preexisting hypertension might experience tachycardia following high doses of bromelain [78]. Moreover, bromelain can cause IgE-mediated respiratory allergies of both the immediate type and the late phase of immediate type [79].

### 6.3. Escin

Escin is a natural mixture of triterpene saponins extracted from the seeds of* Aesculus chinensis Bgem* or* Aesculus wilsonii Rehd*, which mainly consist of A, B, C and D escin. Experimental evidence suggests that escin exerts anti-inflammatory and antiedematous effects. At present, escin injection has been widely used clinically to prevent inflammatory edema after trauma such as fracture and surgery in China [80, 81]. Few studies have analyzed a possible role of escin in male fertility. A Chinese study, evaluating the role of escin in varicocele-associated infertility, showed that a daily oral dose of 60 mg (30 mg every 12 h) for 2 months improved sperm concentration and caused changes in the diameter of the spermatic vein [82]. This dose is higher than the dose used for the venous insufficiency of other districts (orally: 40 mg daily; or intravenously: 5–10 mg daily). Treatment duration is not well established, although medium- and long-term administration do not influence general tolerance.

Escin causes mild adverse effects with very low frequencies, most of which can be reduced or avoided without the need to administer symptomatic drug after advising the patients to take escin after meal. The most important adverse effects occur when escin is administered parentally. The most common reactions are phlebitis and allergic reactions; phlebitis occurring at the first day after intravenous medication accounts for 70% and can cause physical and psychological pain, which directly affects its clinical use. Escin has little effect on vital signs, blood counts, liver, or kidney function.

## 7. Antioxidants

The protective antioxidant system comprehends enzymatic factors (superoxide dismutase (SOD), catalase and glutathione peroxidase (GPX), nonenzymatic factors and low molecular weight compounds (glutathione, N-acetyl-cysteine (NAC), vitamins A, E, and C, coenzyme Q10 (CoQ10), carnitines, myoinositol (MYO), lycopene, astaxanthin,* Serenoa repens*, etc.), and micronutrients (selenium, zinc, and copper)) which interact each other to ensure an optimal protection against the oxidative stress. A deficiency of one of them may result in a decrease of total plasma antioxidant capacity [23]. The main antioxidants are discussed below whereas others are shown in Table 1.

### 7.1. Superoxide Dismutases

SODs are metalloenzymes that convert superoxide to hydrogen peroxide (H_2_O_2_) and comprehend two intracellular and one extracellular forms. The intracellular form contains copper and zinc in the active center (CuZnSOD). It is encoded by the* SOD1* gene and it is mainly localized in the cytoplasm. The other intracellular form is manganese SOD (MnSOD) that acts in the mitochondrial matrix [23]. Its gene,* SOD2*, is inducible under various inflammatory conditions and the nuclear factor kappa B (NF-kB) appears to be the main factor responsible for its induction. Homozygous* SOD2* gene-deficient mice have severe cardiovascular damage and die soon after birth. No abnormality in the genital tract has been reported in these mice. On the contrary, transgenic male mice that express higher levels of MnSOD are infertile. Since SOD only dismutates superoxide anion into hydrogen peroxide, the resulting H_2_O_2_ may also cause a toxic effect in testicular cells [83].

The extracellular form of SOD (ECSOD), encoded from* SOD3* gene, can be present in a free form or can be connected to the surface polysaccharides. It is structurally similar to the MnSOD, but it has zinc and copper in the active center. It has been shown that erectile function is improved by transferring the SOD3 gene to the penis in aged rats. Scavenging superoxide increases nitric oxide (NO) half-life that results in increased cGMP levels [83].

The main active isoenzymes in the seminal plasma are CuZnSOD (75%) and ECSOD (25%) which probably originate from the prostate [84]. SODs have been observed to play a role in the protection of testicular cells against heat stress-induced apoptosis both in vivo and in vitro [85, 86]. Moreover, SOD activity has been lower in infertile patients than in fertile controls and correlates positively with sperm motility and morphology [87].

Some in vitro studies suggest that SODs may play a role in improving sperm parameters in thawed semen samples. In fact, supplementation with manganese (III) mesotetrakis (N-ethylpyridinium-2-yl)porphyrin chloride (MnTE), a permeable SOD mimetic agent, seems to improve total motility, membrane integrity, and viability of goat semen samples after thawing, the acrosomal integrity and the blastocyst formation rate [88]. However, since MnTE supplementation protects spermatozoa from superoxide but not from H_2_O_2_, the degree of sperm parameters improvement is higher when also catalase is simultaneously added [89]. There are evidences for positive effects of the treatment with SODs also on human spermatozoa. In fact, the supplementation of exogenous SODs at the dose of 400 U/mL to the human sperm suspension prevented the loss of motility and the increase of MDA concentration, thus showing a significant role of SODs for human sperm motility [90]. Actually, SOD is included in some over-the-counter products that contain also other anti-antioxidants (e.g., D-chiro-inositol, zinc, and folic acid), recommended for the treatment of male infertility. There is a lack of studies investigating the effects of SOD oral supplementation on human sperm. Hence, there is not agreement about the dosage. However, the most common therapeutic scheme suggested consists of an oral dosage of 150 UI a day for at least three months.

### 7.2. Catalase

Catalase is a heme enzyme with a centrally located iron atom. It catalyzes the decomposition of H_2_O_2_ into molecular oxygen (O_2_) and water (H_2_O). Its presence has been shown in the seminal plasma and spermatozoa of both humans and rats. The extracellular enzyme is produced by the prostate.

Under physiological conditions, catalase plays a role in the nitric oxide-induced sperm capacitation [23]. Its activity has been associated with low sperm quality [91, 92]. In fact, H_2_O_2_, which increases in case of catalase deficiency or reduced activity, plays a substantial role on sperm motility due to the decrease in membrane fluidity that is important for sperm-oocyte fusion [93]. It is not commercially available.

### 7.3. Glutathione Peroxidase

GPXs reduce hydrogen peroxide and organic peroxides, including phospholipids, using the reduced form of glutathione (GSH) as an electron donor [94]. GPXs protect sperm DNA from oxidative damage, playing a role in the chromatin condensation and mainly in the mitochondrial matrix [23]. GPX-defective expression in human spermatozoa has been reported to be associated with OAT [95, 96].

GPXs are divided into two groups according to the presence of selenocysteine in the enzyme isoform. Since selenium deficiency has been associated with male infertility, selenium-containing GPXs are suspected to be a candidate for the defective molecule. At least four isoenzymes belong to the selenium-containing GPX in mammals.

GPX1 is a cytosolic isoform, widely distributed in tissues and, like other antioxidant enzymes, and prevents apoptosis induced by oxidative stress and other stimuli. GPX1 knockout mice do not show reproductive abnormalities.

GPX2 and GPX3 are gastrointestinal and plasma isoforms, respectively.

GPX4 is highly expressed in testis and represents about the 50% of the capsule material, which embeds the helix of mitochondria in the midpiece of spermatozoa.* GPX4* gene knockout mice show premature embryonal death. The* GPX4* gene encodes also for a protein that has a high sequence identity to GPX4 except for the N-terminal region. This protein is specifically present in sperm nuclei and is considered to act as protamine thiol peroxidase.

GPX5 is a nonselenium enzyme. It is exclusively expressed in the epididymis and secreted in the caput and caudal epididymal lumen. It represents the 6% of the secretory epididymal proteins. However, the activity of nonselenium dependent GPX in low and, hence, its contribution as a GSH-dependent peroxide scavenger is ambiguous [83].

However, neither catalase nor GPXs are currently used for the treatment of male infertility.

### 7.4. Glutathione

Glutathione is a sulfur-containing tripeptide present in both a reduced (GSH) and an oxidative (GSSG) states. GSH participates in preserving the intracellular milieu in a reduced state and, in addition, it is an electron donator to GPX. GSH displays its antioxidant activity by the reconstruction of thiol groups (-SH) in proteins and preventing cell membrane from lipid oxidation [23].

GSH levels are maintained through two metabolic pathways: one is the de novo synthesis from Cys, Glu, and Gly that is catalyzed by the *γ*-glutamylcysteine synthetase (*γ*GCS) and glutathione synthetase; the other is its recycling by glutathione reductase, using NADPH as an electron donor. Glutathione is pumped out when it is oxidized or forms conjugates with cytotoxic compounds, including xenobiotical chemicals. Plasma glutathione is hydrolyzed *γ*-glutamyl transpeptidase (GGT), localized at the cellular surface as a membrane protein, into its consisting amino acids that are then taken up by the specific transporter and reused by cells [83].

In animals, GSH therapy affects positively sperm quality. It has a crucial role in increasing sperm motility and fertilization in bulls with asthenozoospermia due to varicocele and in rabbits with dispermy caused by cryptorchidism [97].

Glutathione reduction in human seminal plasma leads to instability of the midpiece of the spermatozoa, resulting in a motility disorder [98]. Some authors suggested that its supplementation plays a therapeutic role in some andrological diseases, particularly during inflammation. Accordingly, its supplementation in infertile men with unilateral varicocele or inflammation of the urogenital tract (two conditions in which radical oxygen species or another toxic compound production plays a pathogenetic function) leads to a significant improvement of sperm parameters, such as concentration, motility, and morphology [99]. These effects on spermatozoa can be partially reversed in case of not too severe structural cell membrane damage [98], a prominent protective role of GSH on the lipid components of the cell membrane [21].

Glutathione is given at the dosage of 600 mg/day intramuscularly for 2-3 months. Hence, glutathione is seldom prescribed for male infertility. However, because of the route of administration, the compliance is low and, currently, GSH is hardly used for the treatment of male infertility.

### 7.5. N-Acetyl-Cysteine (NAC)

NAC is a glutathione precursor. NAC is effective in metal chelation and it seems to improve sperm motility and to prevent sperm DNA oxidative damage [23]. In an animal model, it is able to improve sperm parameters and seminal vesicles weight, previously altered by treatment with As_2_O_3_ [100]. Furthermore, it significantly improves seminal fluid volume and viscosity and increase sperm motility in humans [101]. In women suffering from polycystic ovary syndrome, NAC (administered at the doses of 1500 mg/day) has been shown to ameliorate oocyte and embryo quality, representing an alternative to metformin prescription [102]. Such findings support the therapeutic use of this compound for the treatment of human infertility.

The most common oral dosage used is 600 mg/day. NAC is commercialized in combination with other antioxidants. Its administration is suggested for at least three months.

### 7.6. Vitamin A

Carotenoids are a group of fat-soluble organic compounds found mainly in yellow, red, orange, and pink vegetables. These retinoids are precursor of vitamin A. Retinal is formed from them in the gastrointestinal tract, before being converted to retinol, the most important component of vitamin A.

Carotenoids are natural antioxidants that protect cell membrane integrity, regulate epithelial cell proliferation, and are involved in the regulation of spermatogenesis [23]. Moreover, in rats retinoids have various effects on fetal and neonatal Sertoli, germ, and Leydig cell [103]. Carotenoid deficiency in the diet can lead to a reduction in sperm motility [23]. Studies conducted on bulls have shown that retinol might stabilize sperm acrosomal membrane when oxidative stress increases because of the high temperature [104]. In humans, lower serum retinol concentration has been correlated to a worse sperm quality [105]. Hence, vitamin A administration could be a therapeutic choice for the treatment of human male infertility. Not many studies have evaluated the effects of vitamin A on human sperm parameters. Currently, it is seldom added to the over-the-counter preparation for male infertility treatment. However it should be strengthening the concept that vitamin A has been shown to be toxic and may be teratogenic in higher dose [106, 107].

### 7.7. Vitamin C (Ascorbic Acid)

Vitamin C has a 10-fold higher concentration in seminal plasma than in the serum [108]. It has a more powerful antioxidant action when peroxyradicals are present in the aqueous phase [109] than in the lipid membrane [110]. In mice, at a concentration equivalent to the human therapeutic dose (10 mg/Kg), it is able to reduce MDA concentration, increasing sperm count and the proportion of normal sperm population [111].

In humans, the seminal acid ascorbic levels correlate positively with the percentage of morphologically normal spermatozoa [112] and negatively with DNA fragmentation index [113]. This finding supports the therapeutic use of vitamin C in infertile males. At concentrations lower than 1000 *µ*mol/L, vitamin C is an antioxidant; at higher concentrations it acts as a prooxidant agent [22, 114]. The dose of 1 g/day provides a 2.2-fold increase in plasma acid ascorbic concentrations [115]. The majority of the studies reported in literature investigating the effect of vitamin C administration on sperm quality refer to this orally administered dosage [116, 117]. Accordingly, 1 g daily vitamin C treatment improves human quality, increasing mean sperm count, concentration, and motility [118]. The duration of the treatment is not well established. There is evidence for sperm parameters amelioration after one month of treatment [117], but longer period of treatment is also reported [116]. The dose of vitamin C must, however, is not excessive because high doses may act as a prooxidant, particularly in persons with the haptoglobin type 2- 2 [106, 107].

### 7.8. Vitamin E

Vitamin E (*α*-tocopherol) is a fat-soluble organic compound mainly localized in cell membranes. It protects sperm cell membrane form oxidative stress-induced damage, preventing lipid peroxidation and capturing free hydroxyl radicals and superoxide [23]. Efforts have been made to establish if sperm parameters could be improved by vitamin E supplementation [119]. Its seminal plasma concentration significantly increases when it is administered at doses of 300 and 1200 mg/day for three weeks [120]. The *α*-tocopherol concentration in spermatozoa is independent from the total *α*-tocopherol concentration in seminal plasma, and it significantly correlates with the percentage of motile spermatozoa [121]. A double-blind placebo-controlled trial showed an improved sperm function in vitro after 600 mg daily administration of vitamin E for three months. The administration resulted in improved sperm motility and ability to bind the hamster oocyte in the hamster egg penetration test [119]. In other studies, lower doses (200 or 300 mg/day) have been used [122–124].

A placebo-controlled double-blind study reported an improvement of sperm motility in men with oligoasthenoteratozoospermia after vitamin E oral supplementation. The enhancement of sperm motility was associated with a decreased sperm production of MDA, the end product of the lipid peroxidation [125]. Furthermore, in the course of the six-month treatment period, 21% of patients belonging to the treated group achieved pregnancy [125]. Seminal plasma MDA decrease seems to correlate with the percentage of successful attempts to achieve pregnancy. In a prospective study, 15 normozoospermic men with low fertilization rates in their earlier ART cycles were treated with 200 mg/day of vitamin E for three months. The high MDA concentration declined to normal rate and the fertilization rate per cycle significantly improved after one month of treatment [124]. Finally, dietary habits seem also to play a role in semen quality, since a positive correlation has been found between vitamin E dietary intake and progressive and total motility [118]. This evidence suggests that vitamin E may have a positive effect on semen quality, enhancing the pregnancy rate. Altogether, these findings suggest that vitamin E could be a therapeutic choice for the treatment of male infertility. However, we would emphasize that, besides their interesting antioxidant properties, vitamin E analogs, especially tocopheryl succinate, can exert adverse effects on gap junctional intercellular communication, which could explain their controversial effects in spermatogenesis [106, 107].

### 7.9. Coenzyme Q_10_

CoQ_10_ is the only lipid soluble antioxidant synthetized endogenously. In the Q-cycle it is present in three redox states: ubiquinone (CoQ_10_-oxidized), ubiquinol (CoQ_10_H_2_-reduced), and semiquinone (partially reduced, as radical). The reduced form has a higher antioxidant effect and concentration in the body is approximately 90% of total CoQ_10_. It inhibits protein and DNA oxidation and lipid peroxidation. CoQ_10_ regulates the mitochondrial electron transport in the respiratory chain, receiving electrons from complex I and complex II and passing them to complex III, and it transfers protons from fatty acids to the matrix. It also regulates the permeability transition pore opening and nutrition uptake through the voltage dependent anion channel (VDAC) of the outer mitochondrial membrane [126].

A number of clinical studies documented the beneficial effects of ubiquinone on male fertility. In fact, it has been shown to improve sperm parameters (concentration, motility, and morphology) in men with idiopathic OAT [127, 128], with positive effects on the pregnancy rate [129]. It displays a protective effect against oxidative stress and sperm DNA damage. In addition, CoQ_10_ treatment improves sperm parameters and antioxidant status in infertile men with varicocele [126]. Different therapeutic schemes are reported in literature. The lower dosage used is 90 mg/day for a minimum of 3 months to a maximum of 9 months, but positive effects on sperm parameters have been obtained also by higher dosages (e.g., 100 mg/day for three menses, 200 mg for six months, etc.). The highest dosage reported is 300 mg twice a day for twelve months [126].

Ubiquinol has a stronger antioxidant action in comparison with ubiquinone. It can regenerate other antioxidants such as vitamin E and vitamin C. In addition, CoQ_10_ testicular biosynthesis is very active and high levels of ubiquinol are present in sperm [130]. Ubiquinol sperm concentration strongly correlates with sperm count, motility, and morphology. In addition, Q_10_-total concentration directly correlates with sperm motility [126]. Ubiquinol is administered orally at a dosage of 150 to 200 mg daily, for at least four months.

Carnitine-ubiquinol combination displays a beneficial effect on sperm mitochondrial function of infertile men. It may be due to their uptake by VDAC of the outer mitochondrial membrane, along with the activity of carnitine palmitoyltransferase I (CPTI) in outer mitochondrial membrane. However, further studies are required to support this hypothesis [126].

### 7.10. Carnitine

Currently, carnitine is the molecule with antioxidant activity that has the greatest consensus in literature, especially in its forms L-carnitine and L-acetylcarnitine.

L-carnitine (mainly of exogenous origin though human beings are able to synthesize) is a high-polar, water-soluble quaternary amine. It acts as an essential cofactor for the transport of long chain fatty acids within the mitochondrial matrix in order to facilitate the oxidative processes and to enhance cellular energy production [131, 132]. L-acetylcarnitine, instead, is formed by the enzyme acetyl-L-carnitine transferase that modulates the intracellular and mitochondrial concentration of coenzyme A (CoA) and acetyl-CoA [10, 133]. Interestingly, high concentrations of carnitine are present in the male reproductive tract and particularly in the epididymis, suggesting its crucial role in energy metabolism and sperm maturation [10, 133]. Hence, carnitine concentration in the ejaculate is considered a marker of epididymal function and some studies have shown a decreased L-carnitine concentration in the seminal fluid of patients with epididymitis [134, 135].

The antioxidant properties of carnitine have been studied in men with male accessory gland inflammation. As previously said, inflammatory processes increase ROS production from leukocytes and/or spermatozoa with a consequent increased oxidative stress. Since prostatovesiculoepididymitis (PVE) is the diagnostic category with a higher level of oxidative stress, some studies have evaluated the antioxidant properties of L-carnitine administration in patients with microbial PVE. The results of these studies have shown that the best effect is obtained administering first antibiotic and anti-inflammatory drug and subsequently L-carnitine. The coadministration of antimicrobial agents and antioxidants is less effective while treatment with L-carnitine alone has no effect [10, 136].

The beneficial effects of L-carnitine on sperm parameters are well known. The data in the literature show a statistically significant improvement of sperm progressive motility in patients with OAT treated with L-carnitine or acetyl-L-carnitine at the dose of 3 g/day [137–139] for some months. Moreover, treatment with acetyl-L-carnitine increases sperm motility and viability in asymptomatic infertile patients with ROS overproduction and ultrasonographic evidence of PVE who already received antimicrobial therapy [140]. However, despite improving sperm parameters, there is no evidence of statistical significant variation of seminal plasma *α*-glycosidase concentration (a marker of epididymal function) and of sperm membrane lipid peroxidation [141]. Combination therapy with L-carnitine (2 g/day) and acetyl-L-carnitine (500 mg twice a day) is also effective in improving sperm quality in infertile patients [142] and it improves the total oxyradical scavenging capacity of the seminal fluid [143] and sperm parameters [144]. Interestingly, the addition of L-carnitine in samples to be cryopreservation improves significantly sperm quality [145].

In conclusion, the administration of carnitines is a rational and effective therapeutic strategy for the treatment of male infertility, since it leads to an improvement in sperm parameters. The best therapeutic scheme is represented by the coadministration of at least 2 g/daily of L-carnitine and at least 1 g/daily of acetyl-L-carnitine for at least three months.

### 7.11. Myoinositol

Inositol is a component of the vitamin B complex. MYO, the most biologically important form in nature, is a precursor of second messengers and it is involved in several signal transduction mechanisms in the cell membrane. It regulates seminal plasma osmolarity, the expression of proteins essential for embryogenetic development and for sperm chemiotaxis and sperm motility. In addition, inositols are involved in sperm capacitation and acrosome reaction.

Incubation with MYO results in an increased sperm motility and in a higher number of spermatozoa retrieved by swim-up in both normozoospermic men and in patients with abnormal sperm parameters. This was associated with an improvement of sperm mitochondrial function in patients with OAT [146, 147]. On this basis, the therapeutic use of MYO has been suggested in both in vivo and in vitro assisted reproductive techniques. Accordingly, oral supplementation with MYO seems to improve sperm parameters [146, 148, 149]. In particular, a double-blind, randomized, placebo-controlled study showed that patients with idiopathic infertility, treated for three months with MYO (2 g twice daily), had a significant increase of sperm concentration, total count, progressive motility, and acrosome-reacted spermatozoa. In addition, MYO rebalances LH, FSH, and inhibin-B concentrations [148].

The most frequently reported supplementation strategy consists of a daily oral dose of 4 g (plus 400 *µ*g of folic acid), for at least two months.

### 7.12. Lycopene

Lycopene is a constituent of the human redox protection mechanism against ROS. Although few studies investigated its effects on sperm parameters, it seems to be a therapeutic choice in the treatment of idiopathic male infertility. In facts, its oral administration (2 g twice a day for 3 months) improved sperm concentration and motility [150]. Furthermore, lycopene displays a protective effect against cryopreservation injury of postthawing human spermatozoa. In fact, the addition of lycopene at a proper concentration to cryoprotectant reduces oxidative damage to sperm mitochondria in the freezing-thawing process, attenuates oxidative stress injury induced by ROS to sperm plasma membrane, and improves the antiapoptosis sperm ability [151]. Various lycopene supplementation studies conducted on both humans and animals have shown promising results in alleviating male infertility by decreasing lipid peroxidation and DNA damage and increasing sperm count and viability. Improvement of these parameters indicates a reduction of the oxidative stress, and thus spermatozoa were less vulnerable to oxidative damage, which increases the chances of a normal sperm fertilizing the egg. Human trials have reported sperm parameter improvement and pregnancy rates with supplementation of 4–8 mg of lycopene daily for 3–12 months. However, further detailed and extensive research is still required to determine the dosage and the usefulness of lycopene for male infertility treatment [152].

### 7.13. Astaxanthin

Astaxanthin is natural occurring xanthophyll. Only one study described its effects on human male fertility. A supplementation with 16 mg daily of astaxanthin in 30 infertile patients resulted in higher sperm linear velocity, better capacity of binding the oocyte in the hamster penetration test, and higher total and per cycle pregnancy rates compared to the placebo group [153]. In a model of obese rats, vitamins A and E and astaxanthin administration was associated with better viability, motility, and morphology of spermatozoa obtained from the tail of the epididymis and with a significantly higher number of spermatogonium and Sertoli cells at the histological evaluation [154]. This finding led to the hypothesis that low sperm quality of obese men may be improved by the administration of this cocktail of antioxidants. However, other studies on a greater number of patients are needed to confirm if human sperm parameters may benefit from astaxanthin administration. Astaxanthin is administered in combined therapies at a daily dose of 16 mg. The duration of the treatment is not defined; in the mentioned study, astaxanthin was administered for three months [153].

### 7.14. *Serenoa repens*

*Serenoa repens* (saw palmetto) is a natural product with anti-inflammatory properties, derived from the American dwarf palm [155]. Currently, it is used to treat inflammatory symptoms in patients with benign prostatic hyperplasia and chronic prostatic inflammation [156]. Up to now, only a few and old studies have investigated its effects on sperm parameters. According to Ondrizek and colleagues, high concentration of* Serenoa repens*,* Echinacea*, or ginkgo inhibits human sperm motility [157, 158]. Moreover,* Serenoa repens* does not have any effect on oocyte penetration and sperm DNA integrity, while other herbs (such as* Echinacea purpurea*,* Ginkgo biloba*, and* Hypericum perforatum*) damage these parameters [157, 158]. On this basis,* Serenoa repens* does not seem to ameliorate sperm parameters. Further studies are needed to confirm this conclusion.

### 7.15. Micronutrients

The concentration of micronutrient such as selenium, zinc, and copper correlates with human sperm quality [23].

#### 7.15.1. Selenium

Selenium is a micronutrient essential for normal testicular development, spermatogenesis, sperm motility, and function. The lack of selenium has been correlated to seminiferous epithelium atrophy, disorders of spermatogenesis, maturation of spermatozoa in the epididymis, testis volume reduction, decreased sperm motility, and altered sperm morphology (mainly in the head and in the midpiece) [23, 159]. The exact mechanism by which selenium reduces oxidative stress and improves sperm parameters is still controversial. Its action seems to be mediated by selenoenzymes, such as GPXs [160].

Selenium is given orally at a dosage that ranges from 80 *µ*g to 300 *µ*g once a day, alone or in combination with antioxidants, for at least three months.

#### 7.15.2. Zinc

Zinc is a component of over 200 enzymes involved in the biosynthesis of nuclear acids, proteins, and the process of cell division [23]. It has been reported to normalize oxidosensitive indices and catalase-like activity in the seminal fluid of asthenozoospermic patients [161].

Zinc is given orally at the dose of 220 mg once or twice a day for three to four months, alone or in addition to folic acid (5 mg daily). In combined therapies, it is administered at the dose of at least 10 mg a day. It should be given in the esterified formulation as zinc picolinate that is best taken up by the intestinal tract [106, 107].

#### 7.15.3. Copper

Copper is a trace element whose seminal plasma concentration correlates with sperm quality [23]. A few studies report a positive effect of copper supplementation on sperm parameters in animals. In vitro supplementation with copper on ejaculated buffalo sperm led to a better protection through the process of dilution, equilibration, and freeze-thawing at the dose of 32 *µ*g/L compared to the dosage of 64 *µ*g/L [162]. In addition, dietary copper supplementation at a dosage of 110 mg/kg has been shown to prevent the adverse effects produced by 12 mg/kg/day of tetrathiomolybdate in rat spermatozoa [163]. No studies have explored the effects of copper supplementation on human sperm parameters. Copper is not prescribed for the treatment of male infertility.

#### 7.15.4. Combined Therapies

The synergic effect of some component with different antioxidant properties has been studied. In a randomized trial, the daily administration of vitamin E (400 mg) and selenium (225 *µ*g) for three months resulted in a significant decrease in MDA concentrations and an improvement of sperm motility [93]. In mice, the coadministration of vitamin C (10 mg/kg) and vitamin E (100 mg/kg) led to a decrease of testicular MDA content, along with increased sperm count and decreased percentage of spermatozoa with abnormal form [111]. According to Greco and colleagues, the daily administration of vitamin C (1 g) and vitamin E (1 g) for two months significantly decreased the percentage of human DNA-fragmented spermatozoa [164, 165]. In addition, this association led to an improvement in the clinical pregnancy and implantation rates compared to the group of patients receiving placebo [164, 165]. Furthermore, there is evidence for an increase of seminal vitamin E concentration and of its oxidative stability after cryopreservation if high amount of dietary *α*-tocopheryl acetate is consumed. A decrease of seminal plasma ascorbate concentration is associated with a decreased fertilization rate. In rabbits, a high amount of dietary vitamin E (50 versus 200 mg/kg diet) significantly increased the level of vitamin E in the seminal fluid and the sperm oxidative stability after storage at 5°C for 24 h.

Ascorbic acid showed a different effect in relation to the vitamin E status of animals: when associated with the higher level of vitamin E it increased vitamin E and the semen oxidative stability, whereas both parameters were reduced with lower vitamin E levels. Hence, their combination significantly improves the viability and kinetics of spermatozoa with an increased fertility rate [166]. These evidences sustain the role of the coadministration of vitamins E and C for the improvement of sperm quality in idiopathic male infertility.

The use of NAC, vitamin A, vitamin E, and essential fatty acids in 27 infertile males increases sperm count and decreases ROS and 8-hydroxydeoxyguanine concentration in oligozoospermic patients. Moreover, the treatment improved the acrosome reaction rate and the proportion of PUFA in phospholipids and sperm membrane [167]. In addition, a 26-week-long daily administration of selenium (200 *µ*g) and NAC (600 mg) in 468 infertile men with idiopathic asthenozoospermia resulted in an improvement of all sperm parameters. Furthermore, a positive relationship between seminal plasma concentration of NAC and selenium and sperm parameters has been found [168].

List of all available treatments and other antioxidants is shown in Tables 2 and 3.

## 8. Other Treatments

### 8.1. Pine Bark Extract

The extract of the bark of the (Mediterranean) pine tree,* Pinus maritima*, is rich in polyphenols, namely, anthocyanidins with antioxidant effect, and it reduces inflammatory reaction through the inhibition of the Cyclooxygenase (COX) enzymes 1 and 2 [106] and of the Nuclear Factor Kappa B (NF-kB). Its potential use can be of interest in order to counteract sperm oxidative stress.

### 8.2. *Lepidium meyenii*

*Lepidium meyenii*, also called maca, is another plant belonging to the cruciferous (Brassica) family, growing in the Peruvian Andes Mountains. It has been considered phytoadaptogen that increases the production of the Heat Shock Protein P 72, by reducing the negative impact of stress on protein conformation and cell death [106]. Moreover, the potential increase of sexual desire induced by this plant without influencing testosterone concentration in adult men could be of importance for male infertility.

### 8.3. Polyunsaturated Fatty Acids

Polyunsaturated fatty acids have been recommended for nutrition thanks to their several benefits. In fact these fatty acids improve cell membrane fluidity and function, may possibly protect against cardiovascular diseases, and regarding male infertility may be potentially useful for the spermatogenesis [106, 107].

## 9. Summary

The identification of etiopathogenetic mechanism(s) allows clinicians to select the optimal treatment to overcome male infertility. The therapeutic repertoire includes antibiotics, anti-inflammatory drugs, antioxidants, and micronutrients. Antibiotics are prescribed when an urogenital infection is identified. The most used antibiotics in clinical practice are quinolones (ciprofloxacin, levofloxacin, etc.), tetracyclines, macrolides, trimethoprim, and *β*-lactam antibiotics (penicillin derivatives, cephalosporins, monobactams, and carbapenems). Anti-inflammatory drugs should be given when leukocytospermia and/or inflammatory sign and/or symptoms are present. Finally, antioxidants and micronutrients may be used to protect spermatozoa from oxidative stress overproduction that occurs in many clinical conditions. This class includes a vast array of molecules that may be prescribed alone or in combination. In conclusion, many nonhormonal compounds are available for the treatment of the infertile patient. This allows a customized therapeutic strategy.

## Figures and Tables

**Table 1:** 

Antibiotic	Eradication rate	References
Ciprofloxacin	40–77%	[11, 12]
Levofloxacin	75%	[11]
Azithromycin	80%	[12–14]
Doxycycline	77%	[14]
Clarithromycin	80%	[13]
Azithromycin + ciprofloxacin	62–77%	[4]

**Table 2:** List of all available treatments.

Antibiotics	Quinolones, trimethoprim, tetracyclines, macrolides, *β*-lactam antibiotics
NSAIDs	Salicylates, profens, sulfonanilides

Steroidal anti-inflammatory drugs	Docosanoic acid (DHA), eicosanoid acid (EPA)

Fibrinolytic treatment	Serratiopeptidase, bromelain, escin

Antioxidants	Superoxide dismutases, catalase, glutathione peroxidase, glutathione, N-acetyl-cysteine, vitamin A, vitamin C (ascorbic acid), vitamin E, coenzyme Q10, carnitine, myoinositol, lycopene, astaxanthin, *Serenoa repens*

Micronutrients	Selenium, zinc, copper

**Table 3:** Other antioxidants.

Amino acids	Arginine, taurine, ornithine, citrulline
Vitamins	Vitamins of group B complex, niacin (vitamin PP), pantothenic Acid, folic acid

Omega-3 fatty acids	Docosanoic acid (DHA), eicosanoid acid (EPA)

Others	Magnesium, flavonoid, *Curcuma longa*, *Camellia sinensis*, *Urtica dioica*, *Lepidium meyenii *Walp., Muira Puama(*Ptychopetalum olacoides *Benth), *Ginkgo biloba*, *Scutellaria baicalensis*, *Georgi radix*, *Pinus massoniana*, *Cucurbita maxima*, *Aesculus hippocastanum*, *Crocus sativus*, *Epilobium (angustifolium* and *parviflorum)*, *Citrus bergamia*, *Orthosiphon*, etc.
